# Spinal muscular atrophy type III complicated by spinal superficial siderosis: a case report with molecular and neuropathological findings

**DOI:** 10.1186/s40478-020-01063-9

**Published:** 2020-11-09

**Authors:** Catherine Elizabeth Pringle, Robert Nelson, Willie Miller, Rashmi Kothary, Jean Michaud

**Affiliations:** 1 Division of Neurology, Department of Medicine, The Ottawa Hospital, University of Ottawa, Ottawa, Canada; 2 Department of Diagnostic Imaging, The Ottawa Hospital, University of Ottawa, Ottawa, Canada; 3 Regenerative Medicine Program, Ottawa Hospital Research Institute, Ottawa, ON K1H 8L6 Canada; 4 Department of Cellular and Molecular Medicine, University of Ottawa, Ottawa, ON K1H 8M5 Canada; 5 Department of Biochemistry, Microbiology, and Immunology, University of Ottawa, Ottawa, ON K1H 8M5 Canada; 6 Department of Medicine, University of Ottawa, Ottawa, ON K1H 8M5 Canada; 7 Centre for Neuromuscular Disease, University of Ottawa, Ottawa, ON K1H 8M5 Canada; 8 Department of Pathology and Laboratory Medicine, University of Ottawa, Ottawa, Canada

**Keywords:** Spinal muscular atrophy type III, Superficial siderosis, Sensory symptoms, Radiculo-myelo-neuropathy, Neuropathology, Scoliosis, Dural defects

## Abstract

Spinal muscular atrophy (SMA) is largely linked to deletion or mutation of the Survival motor neuron 1 (*SMN1*) gene located on chromosome 5q13. Type III (Kugelberg–Welander disease) is the mildest childhood form and patients may become ambulatory and have a normal life expectancy. We report the clinical history and morphological findings of a 55-year-old woman who began to experience motor problems at the age of two. She was never fully ambulatory, and her severe scoliosis required the insertion of surgical rod at age 19. Unexpectedly, around 35 years of age, she began to experience sensory symptoms best characterized as a myelo-radiculo-neuropathy with pain as the dominant symptom. Investigations never clarified the etiology of these symptoms. Molecular confirmation of SMA type III was done post-mortem. Neuropathological examination showed classic changes of lower motor neuron neurodegeneration, in line with those reported in the single molecularly confirmed case published so far, and with findings in rare cases reported prior to the discovery of the gene defect. A key autopsy finding was the presence of a severe superficial siderosis of the lower half of the spinal cord. In recent years, the concept of duropathy was put forward, associating superficial siderosis of the spinal cord with various spinal abnormalities, some of which were present in our patient. The presence of significant hemosiderin deposits in the spinal cord and sensory nerve roots with associated tissue and axonal damage provide a plausible explanation for the unexpected sensory symptomatology in this mild lower motor neurodegeneration.

## Introduction

Spinal muscular atrophy (SMA) is largely linked to deletion or mutation of the *Survival motor neuron 1* (*SMN1*) gene located on chromosome 5q13 [15]. This autosomal recessive condition is clinically classified based on the age of onset and the highest motor function achieved. With onset in childhood, three main types are recognized: type I (Werdnig-Hoffman disease), type II (intermediate form) and type III (Kugelberg–Welander disease), first reported in 1956 [9]. Type III is the mildest form with an onset generally occurring in the second year of life. Patients may become ambulatory and have a normal life expectancy [15]. We report a patient with a molecularly confirmed SMA type III whose late clinical history was significantly altered by a superficial siderosis involving the lower spinal cord.

## Case presentation

This 55-year-old woman was born of Anglo-Irish parents following a normal pregnancy and delivery. No neurological disease was known in the family. She learned to sit, roll over and crawl at a normal age and could stand holding onto furniture, but never walked independently. A diagnosis of Kugelberg–Welander form of SMA was proposed when she was 5 or 6 years of age. A single Harrington rods was inserted at age 19 for severe scoliosis, extending from the left T4 lamina to the right S1 lamina. She was integrated into the mainstream school system and went on to obtain a degree in Political Science and a degree in Law. She worked full time until 3 years before her death. She was married at age 30 and despite her severe physical impairment went through two pregnancies, delivering healthy sons.

The following is an extract of the clinical note written at the time by one of the co-authors (RN):

*…diminutive but bright and alert lady whose head is normal in size, but from the neck down has marked under*-*development of her limbs and has a short trunk with marked kyphoscoliosis. Her cranial nerves are all completely normal and she has normal gag and phonation.*

*On motor examination there is rather marked smallness of the neck muscles including the sternomastoids & trapezii. When lying down she cannot lift her head off the pillow and cannot turn her head. However, when she is seated in a wheelchair, she is able to turn her head quite well, although not against any resistance. There is marked weakness of deltoid muscles (1/5) and both triceps & biceps will only function in the plane of gravity. Wrist dorsiflexors are grade 3/5 and handgrip is 3/5. Finger abductors are fairly strong (4/5) but there is extension of the IP joints and she is unable to extend the fingers of the right hand.*

*The lower extremities are held in a position of flexion contraction and it is not possible to fully extend either the knees or the hips. Her right foot is held in a position of inversion and plantar flexion, but the left foot is mobile at the ankle. She has some dorsiflexion movements of the left foot.*

*DTR’s are absent throughout and the left plantar is equivocal. The right plantar is not obtainable. There is a suggestion of a LLQ abdominal reflex but the others are absent. It is noted that the hands and feet sweat normally, but that the fingernails are quite dusky. She does not appear to be dyspneic at this time. No sensory abnormalities are noted but the feet are exquisitely sensitive to touch and pain. Both feet are icy cold…*

She decided to carry on with her pregnancy but, during her second pregnancy, she began to present mild radicular sensory symptoms which resolved after delivery. However, these recurred at 35 years of age, in the right leg and, five years later, in the left one. The pain was initially controlled with morphine. These sensory symptoms increased over the years and were associated with progressive numbness and tingling of the legs, more on the left side. At age 45, she presented with a sudden escalation of pain, at times severe, characterized by burning, dysesthesias and allodynias. Some pain position-sensitive episodes were suggestive of a mechanical etiology. Around the same time, she began to complain of urinary problems with absence of feeling when the bladder was full. On physical examination, she had marked atrophy of all limb muscles, essentially no movement of the lower extremities and very limited movements of the hands (3/5) and proximal upper extremities (2/5). She was globally areflexic with mute toes. The sensory examination showed altered sensation in the L5–S5 dermatomes, in a saddle distribution.

Over a period of three years, mainly to eliminate the possibility of presence of a tumor, two Magnetic Resonance Imaging (MRI) and one Computerized Tomography (CT) of the spine were performed. These were significantly difficult to assess because of the scoliosis and the surgical rod, with skewed images at various angles. They showed marked ectasia of the dural tube from L1 to L3 with marked remodeling of the lamina at the involved levels. There was no evidence of tumor. Retrospectively, hemosiderosis could be detected in a few planes.

A percutaneous endoscopic gastrostomy tube was placed 4 years prior to death, as difficulties with chewing and swallowing, which began at around 35 years of age, became too severe. At age 54, she presented with a severe unrelenting pain in sacral distribution, requiring aggressive treatment, culminating, 7 months later, in an acute pain crisis requiring admission to the intensive care unit. The following years were dominated by respiratory failure requiring a tracheotomy and multiple nosocomial infections. She was found apneic while admitted for a gastrostomy site infection and fistula.

## Materials and methods

The representative sections of the central and peripheral nervous systems and the skeletal muscles were stained with hematoxylin–eosin or hematoxylin-phloxin-safran. Selected sections were stained with one or more of the followings: Masson trichrome, Movat pentachrome, periodic-acid-Schiff (PAS), PAS with diastase, Luxol Fast Blue/PAS (LFB/PAS), Gram, Grocott and iron stain. By immunohistochemistry, the following antibodies were used: glial fibrillary acidic protein (GFAP), neurofilament 200 (NF200), S-100 protein, ubiquitin, α-synuclein and CD-68. Solochrome cyanin was used of frozen peripheral nerve and the Gomori trichrome on frozen skeletal muscle.

A skeletal muscle sample was submitted for analysis for deletions and duplications of exons in *SMN1* and *SMN2* genes using the multiple ligation probe amplification (MLPA) technique.

### Autopsy findings

The general autopsy findings included: low body weight (42 kg), scoliosis with surgical rod, contractures of all joints, focal bronchopneumonia, percutaneous gastric fistula, and severe osteoporosis. There was no significant finding in the heart.

### Neuropathology

#### Gross examination

The brain weighed 1260 grams. The cerebral hemispheres, brainstem, cerebellum, cranial nerves, and blood vessels were unremarkable except for a subtle xanthochromia of the olfactory bulbs and right inferior cerebellar arachnoid. The cut sections showed only a yellow circumscribed lesion, measuring 0.6 cm, in the subcortical region of the left middle frontal gyrus.

**Fig. 1:**
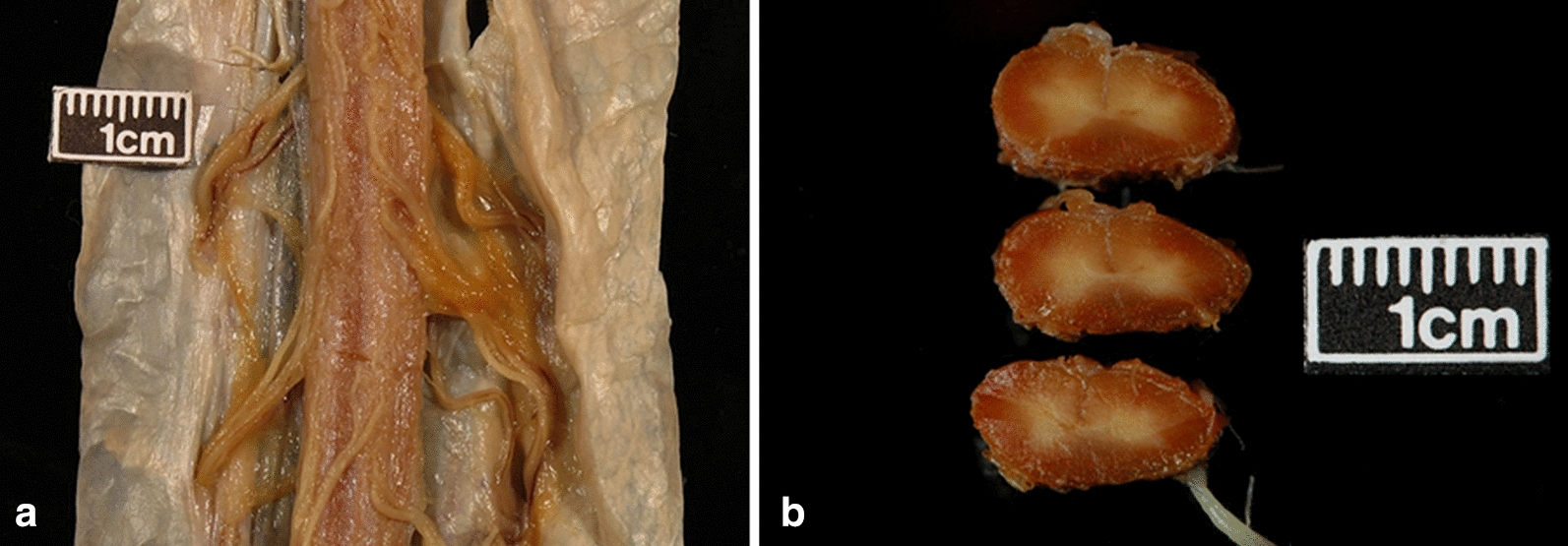
Spinal cord, gross findings. **a** External view of the anterior spinal cord, lower cervical level: presence of very thin anterior nerve roots when compared to the posterior nerve roots; at that level, the superficial siderosis was mild and patchy, mostly involving the nerve roots. **b** Cut sections of the thinned lumbar spinal cord: severe siderosis penetrating deeply the periphery of the spinal cord

The spinal cord was difficult to remove because of numerous adhesions and being thinner than normal. The anterior nerve roots were very thin (Fig. 1a). A striking xanthochromic color was seen in the lower half, being focal and more subtle in the upper thoracic and cervical levels where it involved predominantly the nerve roots (Fig. 1a). The cut sections of the lower spinal cord showed a circumferential superficial xanthochromic rim measuring up to 2 mm in thickness (Fig. 1b).

#### Histology

**Fig. 2:**
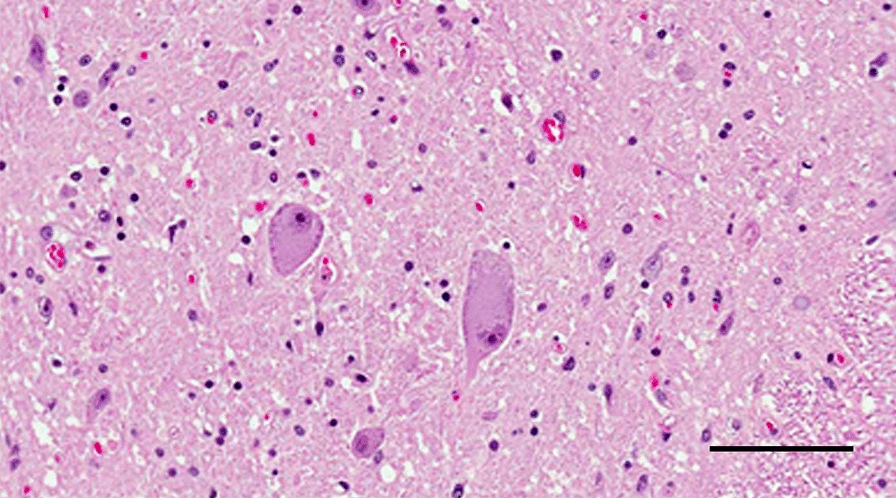
Histology, spinal cord, cervical level. Anterior gray horn showing loss of neurons, two prominent chromatolytic neurons and dense gliosis (Scale bar: 100 μm)

**Fig. 3:**
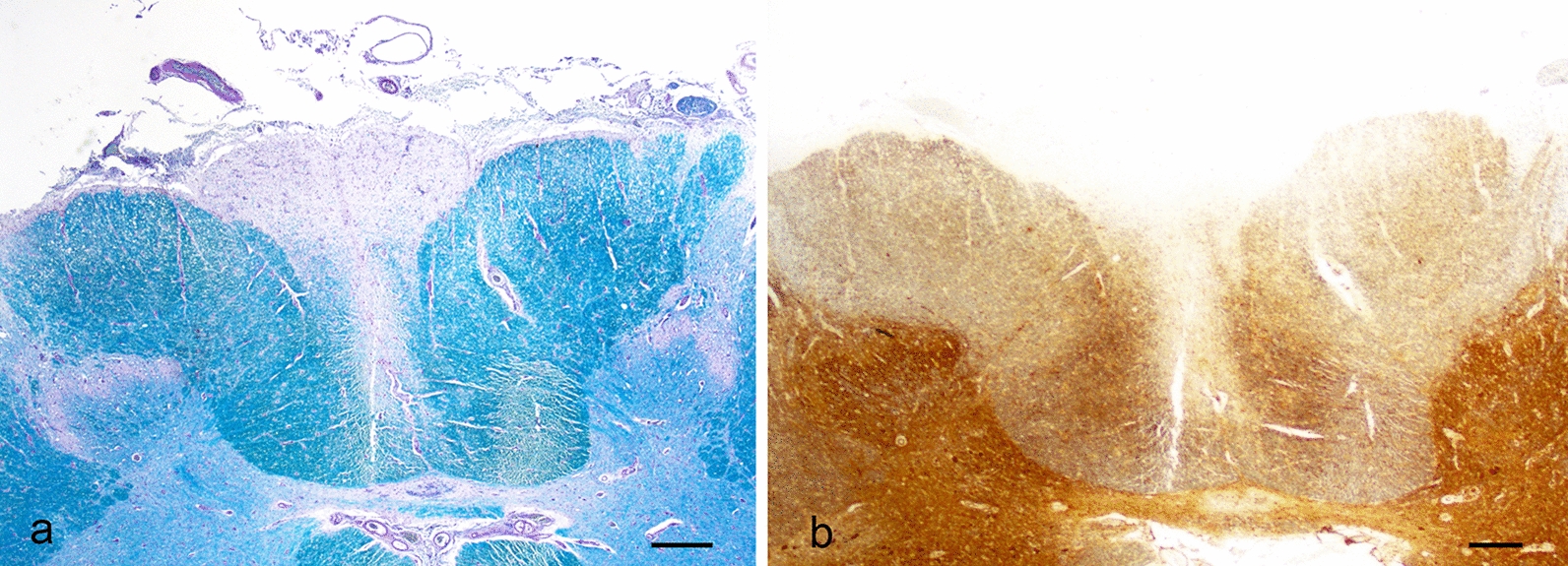
Histology, gracile fasciculi. **a** Low power view of LFB/PAS stained cervical spinal cord showing a pallor of the gracile fasciculi myelin (Scale bar: 500 μm). **b** The NF200 of the same level shows a loss of axons. The outer portion is severely involved, when compared to the middle and inner portions, reflecting the severity of the superficial caudal damage by the hemosiderosis (Scale bar: 500 μm)

In the spinal cord, the anterior gray horns were significantly decreased in volume. There was a severe loss of motor neurons. A few persistent neurons showed chromatolysis (Fig. 2) or, more often, simple atrophy. These changes appeared evenly distributed at all level. There were no intracytoplasmic inclusions and no neuronophagia. Occasional proximal axonal swellings were present, well seen with the NF200. Similar but milder changes were seen in the thoracic nuclei including rare chromatolytic neurons. A very severe GFAP positive gliosis blurred the limit between the gray and white matter. In the upper thoracic and cervical levels, the LFB/PAS stain showed a pallor of the gracile tracts, severe in the outer portions (Fig. 3a), secondary to axonal loss demonstrated with the NF200 (Fig. 3b).

**Fig. 4:**
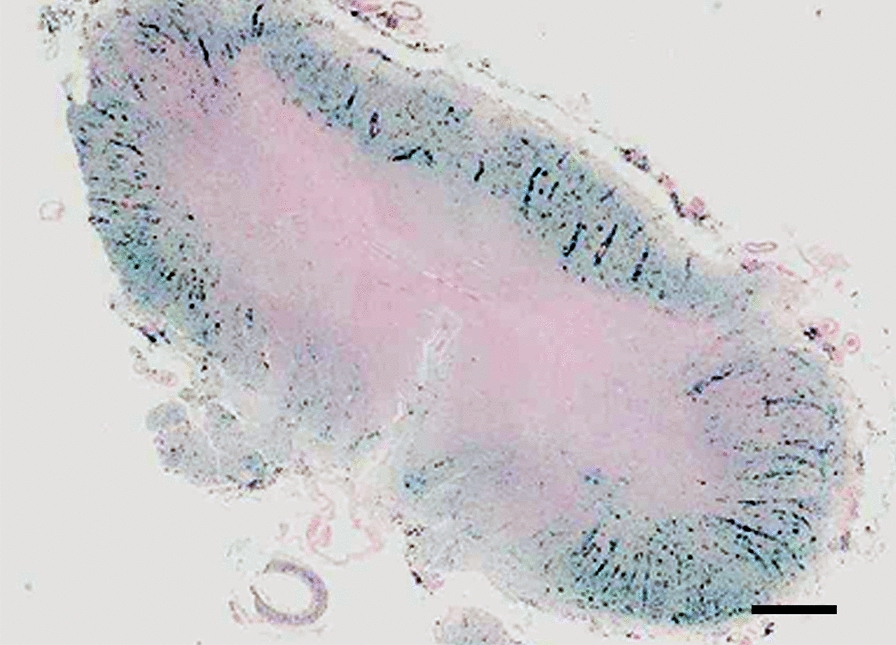
Histology, spinal cord, lumbar level: Very low power view of an iron stain showing a circumferential dense staining except for the para-central ventral regions. The perivascular severe iron accumulation is detected even at this low power view (Scale bar: 1200 μm)

**Fig. 5:**
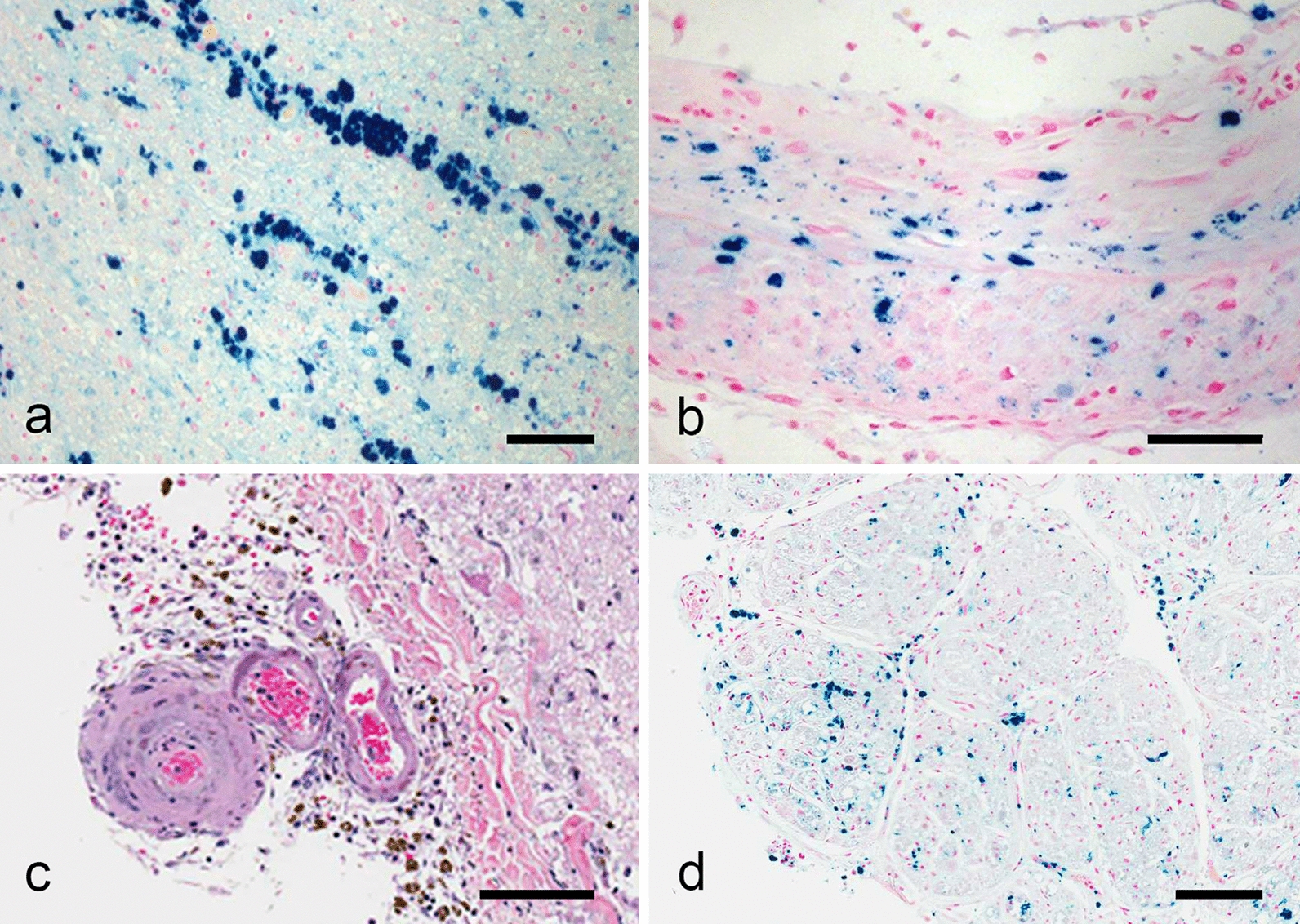
Histology, spinal cord and nerve root. **a** Iron stained of one anterior spinocerebellar tract showing siderophages around blood vessels and in the neuropil (Scale bar: 100 μm). **b** Iron stain showing siderophages and iron granules in all layers of the anterior spinal artery (scale bar: 50 μm). **c** Small leptomeningeal arteriole with thickened and hyalinized wall. Focal calcifications are noted in the venules. Numerous siderophages and fibrosis in the leptomeninges (Scale bar: 50 μm). **d** Iron stain showing siderophages in the endoneurium and perineurium of one posterior nerve root (Scale bar: 100 μm)

The spinal lower thoracic and lumbo-sacral levels showed a severe circumferential hemosiderosis (Fig. 4) characterized by numerous siderophages predominantly in the superficial nervous tissue and along the penetrating blood vessels (Fig. 5a), free iron-positive granules, hemosiderin in astrocytes, severe loss of NF200 reactive axons, occasional axonal swellings, pallor of the myelin, CD-68 positive macrophages and reactive gliosis which included granular astrocytes. In the sacral segment, the hemosiderosis became confluent, obliterating the normal architecture and associated with loss of neurons. The arachnoid showed a diffuse moderate fibrosis with obliteration of the anterior fissure along with numerous siderophages. Several blood vessels including the anterior spinal artery showed iron deposits and siderophages in their walls (Fig. 5b). However, several arterioles showed hyalinization with stenosis and, rarely, obliteration of the lumen along with iron deposition; minute calcifications were also noted in a few venules (Fig. 5c).

The anterior nerve roots were very thin with severe loss of myelinated axons (large ones totally absent) and fibrosis. This loss appeared worse at the level of hemosiderosis. There were a moderate number of GFAP positive glial bundles. The posterior nerve roots appeared to show a subtle loss of axons. There were no glial bundles. At the lumbar level, siderophages were seen in the endoneurium and perineurium of the anterior and posterior nerve roots (Fig. 5d). The dorsal root ganglia showed mild loss of neurons, with reactive satellite cells without definite well-formed Nageotte`s nodules.

In the brainstem, mild to moderate changes, like those described in the anterior gray horns were found in the Vth, VIIth, ambiguous and XIIth cranial nerve nuclei, the latter with rare chromatolytic neurons. No definite change could be found in the other motor cranial nerve nuclei, in the gracile and cuneate nuclei, in the substantia nigra or any of the white tracts including the cerebellar peduncles. The dentate nuclei and Purkinje cells were normal. There was no parenchymal hemosiderosis.

The thalamus did not show any abnormalities. The neurons were within normal limit. The motor cortex showed a normal number of motor neurons (Betz) without degenerative changes. The left middle frontal subcortical lesion corresponded to a largely atretic cavernous hemangioma. There were several siderophages in the surrounding gliotic nervous tissue, but none in the regional leptomeninges.

The biceps and gastrocnemius muscles showed an end-stage picture with massive fatty replacement. Most skeletal muscle fibers were very atrophic with numerous nuclear clumps. When larger, the fibers were variable in size with normal sarcoplasm. The muscle spindles were unremarkable. The peripheral nerves showed subtle loss of myelinated fibers.

The rest of the neuropathological examination disclosed a very mild subacute leptomeningitis with Gram positive cocci, predominant in the spinal canal, and a few tiny remote infarcts in the cerebellar vermis.

In conclusion, the neuropathological examination was consistent with the clinical diagnosis of spinal muscular atrophy type III (Kugelberg–Welander). One significant incidental finding was a severe superficial siderosis involving predominantly the lower thoracic and lumbar-sacral spinal cord and nerve roots.

#### Molecular testing

The molecular analysis (MLPA) done on a sample of skeletal muscle revealed a homozygous deletion of exon 7 of the *SMN1* gene, confirming the clinical diagnosis of SMA. The *SMN2* dosage done three times has reproducibly shown three copies of *SMN2* exon 8 and two copies of *SMN2* exon 7 suggesting this patient had two complete copies of *SMN2* and one copy of likely an incomplete or deleted *SMN2*.

## Discussion

The clinical history of this 55-year-old lady is in line with the diagnosis of SMA type III. She presented her first symptoms before age 2 and the diagnosis was proposed when she was 5 or 6. Interestingly, she was born 1 year before the original description of the mild form of SMA [9]. Although she never walked independently, she had a remarkable life, attaining university degrees and an active professional career. She delivered, against all odds, two children, neither of whom show any sign of a neurodegenerative disorder. The post-mortem molecular testing confirmed the clinical diagnosis of SMA type III although several questions were raised by the development, later in life, of radicular sensory symptoms dominated by pain. Clinically, the diagnosis of SMA type III was never put in doubt but the possible etiology of the radicular symptoms remained puzzling until her death.

As the basic pathology in SMA type III involves the lower motor system, sensory symptoms are not anticipated, and one must consider a second disease or that the sensory symptoms are secondary to the effects of the motor deficits. The clinical symptoms were in keeping with a myelo-radiculo-neuropathy. The cause for the sensory symptoms was not immediately apparent. Although she was significantly physically restricted, she did not seem to be suffering from a nutritional deficiency. In the absence of other obvious causes, the pain was felt to be attributable to the physical–mechanical presence of the rod, compressing nerve roots or peripheral nerves within the spinal canal. A review of the literature is challenging as there is no genetic confirmation in several reports. In a review of 382 patients thought to present SMA type III [19], sensory disturbances were reported in only two patients, one with minimal hypoalgesia in the distal part of the limbs. We are not mentioning the other reported by Kennedy for reasons elaborated below. Kugelberg, in a 1975 review, did not address this specifically [10]. In 1989, Winder and Auer reported a case of familial Kugelberg–Welander disease who presented with sensory abnormalities in the legs, severe enough that an initial diagnosis of Charcot-Marie-Tooth disease had been proposed early in the evolution. The neuropathological examination documented changes consistent with SMA type III and a significant loss of neurons in the dorsal ganglia and demyelination of the gracile tracts [22].

Autopsies reports on patients with SMA type III are not numerous. Only one was genetically documented [14], our report representing a second one. The review of the other autopsied cases of presumed SMA type III patients is again challenged by the lack of molecular confirmation. After critical analysis of the clinical history and genetic transmission, five appears to be consistent with SMA type III cases, including the one reported by Welander and cited by Wohlfart [1, 5, 8, 22, 23]. In three instances [1, 5, 23], the neuropathological reports covered essentially only the anterior horns of the spinal cord. We excluded the cases reported by Kennedy and Magee as the clinical and familial histories are quite characteristics of the X-linked spinobulbar muscular atrophy (SMBA/Kennedy’s syndrome) [6]. We also opted not to include the Paulson et al. report as it may be some form of X-linked amyotrophic lateral sclerosis or related late-onset neurodegeneration [20].

Although the morphological changes are very similar in the non-molecularly or molecularly confirmed groups, the following findings are based on the latter group. They are dominated by a moderate to severe loss of motor neurons of the anterior horns of the spinal cord along with a severe gliosis. The persistent neurons show atrophy or, occasionally, chromatolytic changes. Neuronophagia is rare or absent. There are no abnormal cytoplasmic inclusions. The anterior nerve roots are atrophic with glial bundles. The thoracic nuclei show non-specific inflammatory changes and gliosis, associated, in one case, with a mild loss of neurons with rare chromatolytic ones. The gracile fasciculi are showing mild to moderate demyelination (the severe outer involvement in our case was attributed to the effect of the caudal superficial siderosis). There is mild neuronal loss in the dorsal spinal ganglia. The cortical spinal tracts are unremarkable. In the brainstem, mild loss of neurons is noted in the XIIth cranial nerve nuclei in both cases and, in one case, in the Vth, VIIth and ambiguous pairs. The thalami are normal. Kuru et al. attributed the cortical cerebellar changes to a recent and severe hypoxic event while the finding of a grumose degeneration of the dentate nuclei remained unexplained, yet not felt to be part of the SMA type III morphological picture.

Superficial siderosis of the central nervous system (SSCNS) is relatively rare. In patient with supratentorial involvement, a progressive neurological syndrome has been described as slowly progressive neurosensory hearing loss, associated with an ataxia of gait and, less frequently, pyramidal signs and cognitive impairment [16]. With the advent of magnetic resonance imaging (MRI), the diagnosis of SSCNS is readily feasible, even before the presence of clinical symptoms, as the iron deposition in the arachnoid and superficial nervous tissue generate a characteristic hypointensity on T2-weighted imaging [12, 16]. It is caused by chronic or repeated extravasation of blood in the subarachnoid space over an extended period of time along with dissemination of heme by the circulating cerebrospinal fluid [7].The classic sources of the recurrent bleeding have included: neoplasms, vascular malformations, brachial plexus or nerve root injury, previous head or spinal injury and spinal surgery [7, 11, 12, 17]. There is however a significant number of cases where the source of the bleeding cannot be identified [7]. In recent years, with advanced neuroradiological techniques and with the significant increase in the number of patients with dementia, the finding of cortical SSCNS has been recognized as a marker of amyloid angiopathy [3, 16, 18].

At times, as in our patient, the superficial siderosis involves only or predominantly the spinal cord or part of it. Recently, in some of those patients, an association with dural abnormalities in the spine was proposed as a cause of spinal superficial siderosis [13, 21]. The concept of duropathy as a cause of spinal superficial siderosis, introduced by Kumar in 2012 along with a comprehensive review [13], implies the presence of a dural defect in around half of the cases in their series and are associated with the presence of a ventral longitudinal intraspinal fluid-filled collection. In some, a connection with the arachnoid space is demonstrable. Also, some cases have been reported with protruding discs, osteophytes, dural calcifications at times in disc [2, 13], dural ectasia or diverticula and following anterior cervical spine surgery [4]. In our patient, there was significant dural and spinal distortion generated by the severe scoliosis and the post-surgical procedure with the insertion of a surgical rod, from T4 to S1, level of significant hemosiderosis. Our patient had also an ectasia of the L1–L3 dural space with marked remodeling of the laminae. Thus, we believe that the radiological and morphological findings observed in our patient are highly suggestive of a duropathy as the cause of the chronic extravasation of blood in the subarachnoid space and the ensuing superficial siderosis. In our patient, another possible cause of superficial siderosis could be put forward. There was a small cavernous hemangioma in the left frontal white matter; we considered this as an unlikely cause as the lesion was buried in the white matter, was largely atretic and there were no local arachnoid siderophages.

The pathology of superficial siderosis in the central nervous system (CNS) was meticulously analysed and reviewed by Koeppen et al. [7] based on a detailed analysis of the tissues of nine patients with superficial siderosis. They emphasized that only the CNS tissues convert heme from the cerebral spinal fluid (CSF) into hemosiderin. Their findings included extensive deposits of iron and ferritin in siderotic tissues, severe loss of axons but better preservation of axons where the accumulation of iron is less severe and presence of anuclear foamy structures that appear to be axonal swellings. Various techniques including double-labelled immunofluorescence and ultrastructure did not allow a clear understanding on how axons were damaged and if they were even disrupted. The authors felt that the iron-induced neuronal injury was unlikely reversible [7]. In our patient, in line with these findings, axonal damage due to the superficial siderosis was present and severe, better reflected by the significant loss of NF200 axons (data not shown). Axonal swellings in small number were also noted in the affected tissue. In the sensory pathways specifically, the unbalanced demyelination of the gracile fasciculi (superficial regions as opposed to the inner regions) emphasises the loss of axons within the siderotic regions. In the sensory nerve roots, the axonal loss was subtle, but the deposition of hemosiderin was significant (Fig. 5d). We propose that the nerve root involvement, that is axonal loss or chemical irritation, best explain the sensory symptoms our patient presented in the evolution of her SMA type III with the understanding that mechanical factors cannot be totally excluded. In the above-mentioned review [7], vascular changes (Fig. 5c) were not mentioned. These raise the possibility of localized chronic tissue hypoxia as a contributing factor to the pathogenesis of tissue damage associated with superficial siderosis.

## Conclusions

In conclusion, we report the clinical history and morphological findings of a 55-year-old patient with SMA type III who unexpectedly presented, in the evolution of her disease, lower extremity sensory symptoms dominated by pain and consistent with a myelo-radiculo-neuropathy. A critical review of the autopsies performed on patients with SMA type III show that they are few and that our report is only the second with a molecular confirmation. Classical findings of a lower motor neuron degeneration were found, moderate to severe in the spinal cord and mild to absent in the thoracic nuclei and brainstem motor cranial nerve nuclei. Severe gliosis and neuroinflammatory changes were generalized. The anterior nerve roots were very atrophic with glial bundles. The gracile tracts showed a mild demyelination. The thalami were normal.

In our patient, a superficial siderosis of the lower half of the spinal cord was found. Severe scoliosis, implanted surgical rod and lumbar dural ectasia with marked remodeling of the laminae were felt to be linked to the cause of chronic extravasation of blood in the subarachnoid space, in line with the recently proposed concept of duropathy, associating superficial siderosis of the spinal cord with various spinal abnormalities detected by diagnostic imaging. The tissue/axonal damage associated with the presence of hemosiderin and ferritin in the spinal cord and sensory nerve roots was the most likely substratum for the unexpected development of sensory symptoms.

## Data Availability

The clinical data are retrievable in the patient’s chart. The histological material is located in the Department of Pathology and Laboratory Medicine storage room, at The Ottawa Hospital.
